# Exploring Genetic Markers for Cold–Heat Patterns: Integrating Traditional Medicine With Modern Genomic Research

**DOI:** 10.1155/genr/4503515

**Published:** 2025-11-21

**Authors:** Seogyun Jeong, Sanghun Lee

**Affiliations:** Department of Bioconvergence and Engineering, Graduate School, Dankook University, Yongin-Si 16890, Gyeonggi-Do, Republic of Korea

**Keywords:** cold pattern, East Asian medicine, genetics, heat pattern, integrative research, SNPs

## Abstract

**Background:**

Temperature sensitivity has gained considerable attention in the era of precision medicine. This trait has long been used to identify cold–heat patterns (C-HPs), a diagnostic framework in Traditional Korean Medicine that categorizes individuals based on their thermal responses. C-HP helps understand an individual's inherent physical characteristics, which have been shown to be highly heritable and thus shaped by genetic factors. However, genetic markers that are significantly associated with this trait remain scarce. To address this gap, we aimed to identify candidate single-nucleotide polymorphisms (SNPs) based on previous genomewide association studies (GWASs) of related traits.

**Methods:**

Given the limited research directly addressing C-HP, we incorporated genetic studies related to traits such as “Cold” or “Heat,” as well as thyroid hormone, which plays a key role in thermogenesis through the activation of various metabolic pathways. After selecting the SNPs reported in previous GWAS from the GWAS Catalog (EMBL-EBI), we validated these findings using 90 Korean patients reporting C-HP, with statistical significance assessed through residual permutations. Gene set enrichment analysis (GSEA) was performed using the GO Biological Process 2023 dataset to identify the pathways associated with C-HP. Furthermore, we compared our findings with control traits in order to confirm that the observed associations were specific to C-HP-related traits rather than random correlations. Principal component analysis (PCA) was conducted on candidate SNPs from the 1000 Genomes reference dataset to illustrate the ethnic variation for C-HP across five populations.

**Results:**

Of 63 GWAS, we selected 548 SNPs for validation. Ultimately, 20 candidate SNPs associated with heat patterns and 19 candidate SNPs associated with cold patterns were identified. Of the heat-pattern SNPs, 18 were linked to thyroid hormone traits, with key SNPs including rs12409301 (*CAPZB*) and rs12855 (*CDKN2C*). For the cold-pattern trait, 16 SNPs were associated with thyroid hormones such as rs622474 (*PDE4B*) and rs11204752 (*GOLPH3L*). GSEA confirmed notable enrichment in vascular processes for the heat pattern and mitochondrial organization for the cold pattern. The most significant pathway was vascular smooth muscle cell development (*p* value = 1.28 × 10^−5^) in the heat pattern. The clear ethnic differences in C-HP were observed in the PCA of 1000 Genomes populations where East Asian and African populations formed distinct, well-separated clusters.

**Conclusion:**

Our study suggested a total of 39 candidate SNPs as genetic markers for C-HP that are plausible in the context of temperature sensitivity. We hope that our findings will provide a valuable basis for further biological research and potential clinical applications of C-HP.

## 1. Introduction

In recent years, advancements in precision medicine have emphasized the need for individualized treatment strategies, moving away from conventional, standardized, and disease-centered approaches [1, 2]. This shift is largely driven by the increasing ability to analyze vast amounts of genetic and molecular data, leading to a deeper understanding of individual physiological and pathological variations [3]. As our molecular insights expand, cold–heat patterns (C-HPs), a core diagnostic framework in Traditional Korean Medicine, have also begun to evolve, particularly in their foundational concepts [4, 5]. This framework categorizes patient's constitutional characteristics and disease states into cold patterns (CPs) and heat patterns (HPs) for individualized treatment. CPs are characterized by high sensitivity to cold, manifesting as chills, pallor, and hypothermia associated with decreased energy metabolism, while HPs are characterized by high sensitivity to heat, manifesting as fever, flushing, and hyperthermia linked to immune activation and cellular proliferation [6–9]. Historically rooted in subjective assessments, C-HP is now being reinterpreted through modern scientific methodologies, enhancing both the accuracy and efficacy of diagnosis and treatment.

Recent efforts have focused on integrating biomarkers from genomics, proteomics, and metabolomics into the diagnostic framework of C-HP. By doing so, researchers aim to scientifically standardize C-HP, providing objective, data-driven methods for its diagnosis [8, 10, 11]. This integration of traditional practices with contemporary molecular technologies has the potential to bridge the gap between ancient medical wisdom and modern healthcare, paving the way for a more personalized and precise approach to treatment. Among these efforts, genomic research on C-HP has been particularly active. For instance, gene expression analysis in rheumatoid arthritis identified endocrine-related genes as dominant in CP, while immune-related genes were more prominent in HP. Cold-related genes are linked to energy metabolism, contributing to symptoms such as chills and hypothermia [9]. In addition, significant differences in CD4+ T cell gene expression have been observed between C-HP types. CPs have shown higher expression of genes related to Collagen type IV and innate immune activation, whereas HPs have been previously associated with genes involved in cell proliferation, differentiation, and immune regulation [8]. Furthermore, a heritability study of C-HP in twins suggested a genetic influence, with CP exhibiting a heritability of 40% and HP 33% [12].

A recent genomewide association study (GWAS) conducted on the Daejeon Citizen Health cohort in South Korea identified single-nucleotide polymorphisms (SNPs) associated with CP. This study revealed that individuals with specific cold-related genetic traits exhibited increased inflammatory responses. Notably, SNPs in the *SP1* transcript factor, identified as a cold-related genetic marker, regulate the expression of the cold-inducible RNA-binding protein (*CIRP*), which plays a role in inflammation.- [13]. However, the conventional GWAS only identifies SNPs with genomewide significance (*p* value < 5 × 10^−8^), and larger-scale studies are needed for greater statistical power [14]. While the Daejeon Citizen Health cohort included 2000 participants, this sample size lacked sufficient statistical power to detect a broader range of CP-related genetic markers, suggesting that the C-HP-related markers identified so far represent only a limited subset.

Over the past 2 decades, numerous GWAS have been conducted using large-scale biobanks to identify significant SNPs related to various diseases and traits [15, 16]. However, large-scale studies that specifically focus on C-HP in traditional medicine remain scarce. In this study, we identified candidate SNPs associated with C-HP by leveraging C-HP-related traits from previous GWAS and relevant literature, followed by validation using in-house data. Due to the limited research directly addressing C-HP, we broadened our scope to include genetic studies related to traits such as “Cold” or “Heat,” as well as thyroid hormones. Thyroid hormones play a critical role in thermogenesis by activating key metabolic pathways, making them vital for understanding the physiological mechanisms that drive C-HP. Because hypothyroidism and hyperthyroidism align with C-HP, they serve as effective experimental models [17–19]. This approach not only enhances the accuracy of C-HP diagnosis but also contributes to the broader field of precision medicine by offering insights into the genetic factors that influence individual responses to treatment. Ultimately, our findings may pave the way for more personalized and effective therapeutic strategies, bridging the gap between traditional and modern medicine and improving patient outcomes.

## 2. Methods

### 2.1. Study Selection for C-HPs and Reported SNPs Extraction

**Figure 1:**
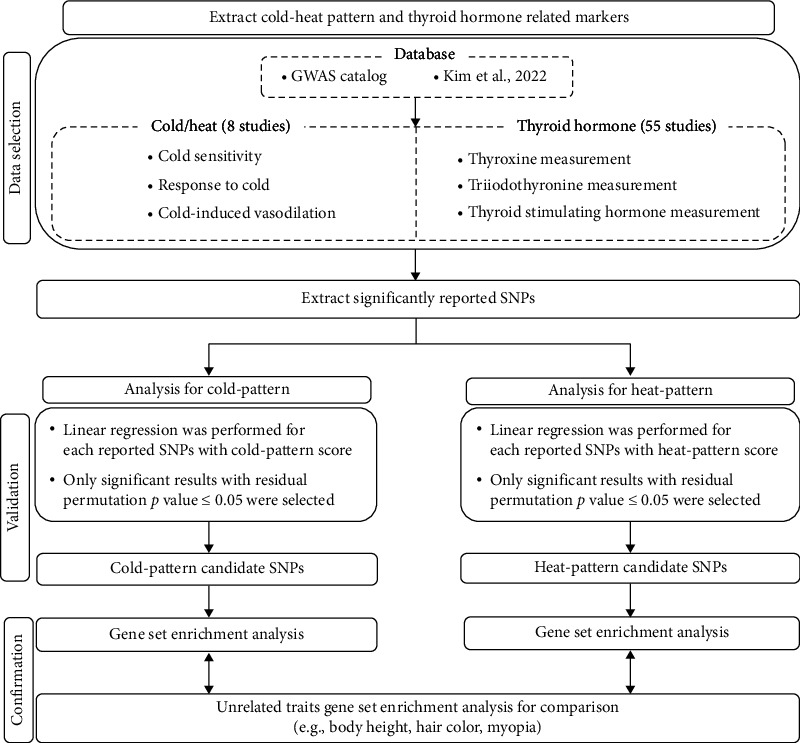
Workflow for extracting and analyzing cold–heat patterns and thyroid hormone markers. Data were sourced from the GWAS catalog and Kim et al. [13]. The reported SNPs were selected and validated using in-house data, leading to the selection of candidate SNPs for cold- and heat-pattern traits. Significant SNPs (*p* value < 0.05) were selected and analyzed using GSEA to explore the related biological processes.

The workflow for extracting and analyzing the identified SNPs related to C-HP is shown in Figure 1. First, we selected reported SNPs from previous GWAS that were associated with traits relevant to C-HP. Next, we validated these SNPs using our own cohort on C-HP and defined candidate SNPs as potential markers for C-HP. The primary sources for identifying these C-HP-related traits were the GWAS Catalog (EMBL-EBI) and an article by Kim et al. (2022) [13].

We searched GWAS studies reporting traits such as “Cold” or “Heat” (cold/heat), as well as thyroid hormones. The cold/heat-related traits included the following: “cold-induced vasodilation” (*n* = 48), “response to cold” (*n* = 81), “*CIRP* measurement” (*n* = 9), and “cold sensitivity” (*n* = 55), with the latter specifically adopted from the study by Kim et al. (2022) [13]. For these traits, a total of 8 studies were found, from which we extracted 128 reported SNPs after excluding X chromosome and overlapping SNPs. The *p* value distribution of these SNPs is shown in Supporting Figure 1A. For thyroid hormone traits, we included the following: “thyroxine measurement” (*n* = 94), “triiodothyronine measurement” (*n* = 34), and “thyroid-stimulating hormone (TSH) measurement” (*n* = 614). For these traits, a total of 55 studies were found, from which we extracted 478 reported SNPs after excluding X chromosome and overlapping SNPs. The *p* value distribution of these SNPs is shown in Supporting Figure 1B.

### 2.2. Validation of Reported SNPs Using In-House Cohort

For validation, we used our 90 patients with direct C-HP assessments measured using the self-administered 15-item C-HP Identification Questionnaire (CHPIQ). It includes eight questions for CP and seven questions for HP [20, 21]. Our analysis was performed using PLINK Version 2.0 (https://www.cog-genomics.org/plink/2.0/) to identify SNPs associated with C-HP. Imputation was performed using the Korean Imputation Service (KIS) provided by the Clinical and Omics Data Archive (CODA, https://coda.nih.go.kr/frt/index.do), utilizing the Korean Phase 1 reference panel (*N* = 4799). Linear regression analyses were conducted for each of the 548 GWAS-reported SNPs available in our in-house dataset with CP-score and HP-score as temperature sensitivity. The regression model was adjusted for potential confounders, including age, sex, and five principal components (PCs). The PCs were generated using the variance-standardized relationship matrix based on a linkage disequilibrium (LD)–pruned set of the variants after pruning with windows of 50 kb, a step size of five markers, and a pairwise *r*^2^ < 0.2. To address the limited sample size, we performed residual permutation testing with 10,000 iterations conducted in R (Version 4.4.1). To identify independent signals, LD-based clumping was performed using PLINK (--clump-p2 1, --clump-r2 0.2, and --clump-kb 250), retaining SNPs for HP or CP, respectively. Only SNPs with permutation-based *p* values < 0.05 were considered statistically significant and retained as candidate SNPs for C-HP.

### 2.3. Gene Set Enrichment Analysis (GSEA) of Genes Linked to Candidate SNPs

GSEA was performed using the GO Biological Process 2023 dataset, with separate analyses for CP or HP genes [22]. Each gene linked to candidate SNPs after validation was identified through GraphQL queries using the Open Targets Genetics API. Enrichment analysis was then conducted using the “enrichr()” function from the gseapy package (Version 1.1.3) with a *p* value cut off of 0.05. The *p* values for the genes were derived from the corresponding SNPs in the GWAS data. The top five pathways for both CP and HP were selected based on the smallest *p* values obtained from the GSEA results. In addition, we conducted pathway similarity network analysis using each trait's top five pathways. Pathway similarity was calculated using the Jaccard coefficient (threshold ≥ 0.3) based on shared genes between pathway pairs. As a control, we applied the same GSEA procedure to traits unrelated to C-HP, for example, body height, hair color, and myopia. This was intended to confirm that the originally selected cold/heat and thyroid traits can serve as reliable proxies for C-HP.

### 2.4. PC Analysis (PCA) of Population Structure

For the candidate C-HP SNPs, ethnical difference was evaluated with the 1000 Genomes reference dataset (https://ftp.1000genomes.ebi.ac.uk/vol1/ftp/release/20130502/), which includes populations from European (EUR) (*N* = 533), East Asian (EAS) (*N* = 539), American (AMR) (*N* = 373), South Asian (SAS) (*N* = 544), and African (AFR) (*N* = 719) groups. The PCs based on the SNPs were calculated using PLINK2.

## 3. Results

### 3.1. Candidate SNPs for HP

Twenty candidate SNPs related to HP were identified and are summarized in Table 1. The full names of all genes mentioned in this study are provided in Supporting Table 1. Among these, 18 SNPs were associated with thyroid hormone traits. In the TSH measurement category, the following SNPs were identified as candidate markers: rs12409301 (*CAPZB*), rs12855 (*CDKN2C*), rs951366 (*PM20D1*), rs59381142 (*HES1*), rs11755845 (*VEGFA*), rs10814915 (*GLIS3*), rs11255790 (*NA*), rs109095868 (*CELF2*), rs4933466 (*PTEN*), rs2475217 (*SLK*), rs643506 (*PPP2R1B*), rs71430783 (*ITPK1*), rs2601803 (*ADCY9*), rs3848445 (*HSS3TB1*), rs75261749 (*UTP18*), rs9915657 (*SOX9*), and rs4552110 (*CCBE1*). Similarly, SNPs associated with thyroxine and triiodothyronine measurements included rs951366(*PM20D1*), rs7045138 (*TRMO*), rs2475217 (*SLK*), and rs3848445 (*HSS3TB1*) for HP. Two additional SNPs were identified in the cold/heat trait category: rs62143197 (*NLRP12*), linked to *CIRP* measurement, and rs4666462 (*OSR1*), linked to a response to cold.

### 3.2. Candidate SNPs for CP

Table 2 summarizes the 19 candidate SNPs associated with CP, of which 16 SNPs are associated with thyroid hormone traits. In the TSH measurement category, the following SNPs were identified: rs622474 (*PDE4B*), rs11204752 (*GOLPH3L*), rs12855 (*CDKN2C*), rs6717283 (*BOK*), rs16874919 (*PPARGC1A*), rs6462411 (*SDK1*), rs1441198 (*SULF1*), rs59282311 (*PDE7A*), rs9298749 (*BNC2*), rs7855088 (*ANP32B*), rs657152 (*ABO*), rs925489 (*TRMO*), and rs7253430 (*ATP8B3*). Additional SNPs included rs6722076 (*UGT1A6*), associated with thyroxine measurement, and rs7020640 (*EGFL7*), which was identified as a candidate for both thyroxine and triiodothyronine measurements. Furthermore, rs6499766 (*LPCAT2*) was associated with thyroxine measurement. In the cold/heat-related traits category, three SNPs were identified: rs1354034 (*ARHGEF3*), linked to *CIRP* measurement; rs17122904 (*PATJ*), linked to cold-induced vasodilation; and rs77101060 (*SORCS1*), linked to cold sensitivity.

### 3.3. GSEA With HP or CP Candidate Genes

**Figure 2:**
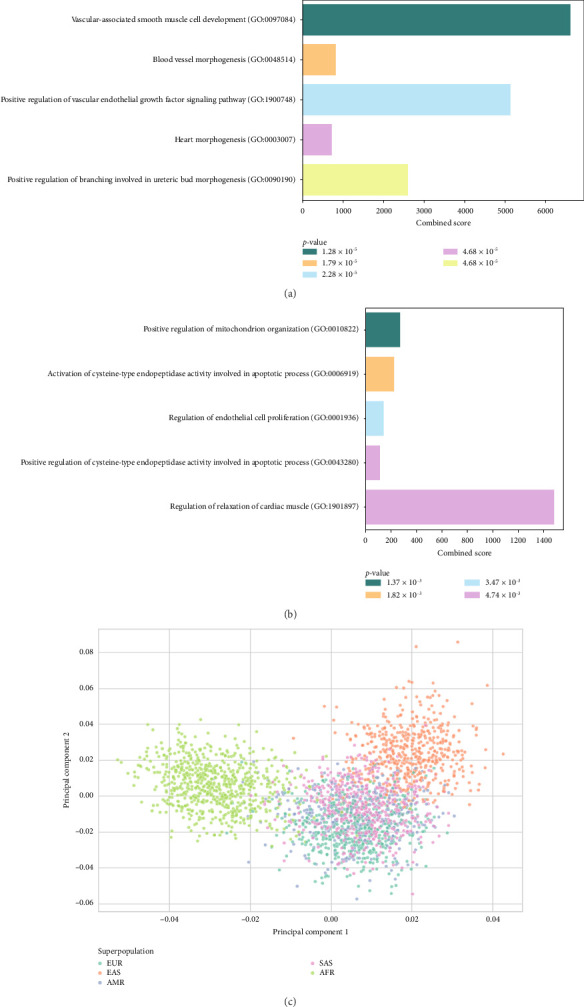
Gene set enrichment analysis (GSEA) results and principal component analysis (PCA) across populations. The *X*-axis represents the combined score and the *Y*-axis lists based on the GO Biological Process 2023 pathways. The top five pathways in each group were selected based on the lowest *p* values. (a) The heat pattern showed significant enrichment in vascular development–related pathways, including vascular smooth muscle cell development and blood vessel morphogenesis. (b) The cold pattern was significantly associated with mitochondrial organization, apoptotic processes, and cardiac muscle regulation. (c) The PCA plot illustrates the genetic clustering using candidate SNPs of 2708 individuals belonging to five populations from the 1000 Genomes dataset: European (EUR), East Asian (EAS), American (AMR), South Asian (SAS), and African (AFR). Each point represents an individual, colored according to population affiliation. The three clusters were distinctly observed along Principal component 1 (PC1) and Principal component 2 (PC2).

The GSEA results for 20 genes related to HP (Figure 2(a)) revealed significant enrichment in biological processes, such as vascular-associated smooth muscle cell development (*HES1* and *VEGFA*), blood vessel morphogenesis (*CCBE1, HES1,* and *VEGFA*), and positive regulation of the vascular endothelial growth factor signaling pathway (*CCBE1* and *VEGFA*). Additional pathways included heart morphogenesis (*HES1, SOX9,* and *VEGFA*) and positive regulation of branching involved in ureteric bud morphogenesis (*SOX9* and *VEGFA*). Among these, the vascular-associated smooth muscle cell development pathway had the lowest *p* value (1.28 × 10^−5^) and the highest combined score (6620), followed by the positive regulation of the vascular endothelial growth factor signaling pathway (*p* value 1.79 × 10^−5^, combined score 5138), indicating a strong relevance to HP. Overall, vascular-related pathways were notably linked to HP. Furthermore, the pathway similarity network demonstrated strong interconnectivity among these five pathways (Supporting Figure 2A).

The GSEA results for the 19 genes related to CP (Figure 2(b)) highlighted significant enrichment in processes such as positive regulation of mitochondrial organization (*PPARGC1A* and *BOK*), activation of cysteine-type endopeptidase activity involved in the apoptotic process (*ANP32B* and *BOK*), and regulation of endothelial cell proliferation (*EGFL7* and *SULF1*). Other enriched processes included the positive regulation of cysteine-type endopeptidase activity in apoptosis (*ANP32B* and *BOK*) and the regulation of cardiac muscle relaxation (*PDE4B*). Among these, positive regulation of the mitochondrial organization pathway had the lowest *p* value (1.37 × 10^−3^) and a combined score of 276, suggesting a potential link between heat production in the mitochondria and CP. In addition, the pathway similarity network showed partial interconnectivity among these five pathways (Supporting Figure 3A).

### 3.4. Pathway Analysis of Control Traits for HP or CP

To assess the specificity of our findings, body height, hair color, and myopia were analyzed as HP and CP using GSEA and pathway similarity analysis. The number of significant SNPs (permutation *p* value < 0.05) and their corresponding mapped genes for each control trait are summarized in Supporting Table 2. Specifically, the combined total of significant SNPs for cold/heat and thyroid traits was slightly higher than all control traits, while they did not individually show the highest significant SNP ratios in either HP or CP. Furthermore, pathway analysis of control traits showed distinct patterns from thyroid-related pathways. In the HP, ISG15-protein conjugation (*UBE2L6*), regulation of transcription by RNA polymerase II (*FOXE1, ZMIZ1, TET2, PAX3, ZNF778, ETV1,* and *EZR*), and positive regulation of epithelial-to-mesenchymal transition (*TCF7L2, TIAM1,* and *CTNNB1*) were the most significantly enriched pathways. In the CP, negative regulation of cellular senescence (*CDK6, HMGA2,* and *TBX2*), melanin biosynthetic process (*OCA2* and *SLC45A2*), and nucleobase-containing compound catabolic process (*RNASEH2C* and *SETMAR*) were the most significantly enriched pathways (Supporting Figures 4 and 5). Pathway similarity analysis revealed limited connectivity between pathways in the HP, whereas the shared genes across CP pathways had no functional relevance to C-HP characteristics (Supporting Figures 2 and 3). Together, these results support that the selected cold/heat and thyroid hormone traits serve as reliable proxies.

### 3.5. Ethnic Variations in C-HP Using PCA

PCA at the individual level revealed clear ethnic differences in C-HP, with distinct clustering patterns observed among the 1000 Genomes Project populations (Figure 2(c)). Each population formed a separate cluster, with EAS and AFR populations showing well-defined and distinct groups. In comparison, the EUR, AMR, and SAS populations displayed considerable overlap in their clustering patterns.

## 4. Discussion

Our analysis identified 39 candidate SNPs significantly associated with C-HP traits. Notably, the majority of these variants were linked to thyroid function. The GSEA results revealed that the HP candidate SNPs were predominantly involved in vascular-related pathways, whereas the CP candidate SNPs were significantly associated with mitochondrial functions. For HP candidate markers, vascular regulation is closely linked to blood flow, which can affect heat sensitivity and potentially contribute to HP [23, 24]. Key genes in these pathways include *HES1, VEGFA, CCBE1*, and *SOX9*. *HES1* and *VEGFA* are crucial for angiogenesis, with *HES1* acting as a key effector in Notch signaling, regulating the arterial specification of vascular smooth muscle cells [25, 26]. *VEGFA* is a well-known angiogenic factor [27], while *CCBE1* modulates the maturation of VEGF-C-stimulating angiogenesis [28]. *SOX9* also plays a key role during vascular development, further reinforcing the link between these genes and HP [29].

For the CP candidate markers, the regulation of mitochondrial organization was significant. Mitochondrial uncoupling is a key factor in body temperature regulation because uncoupling proteins (UCPs) release heat during ATP production [30, 31]. Downregulation of genes associated with candidate CP SNPs may reduce mitochondrial heat production. The key genes in this pathway include *PPARGC1A* and *BOK*. *PPARGC1A* encodes the PGC-1α protein, which induces the expression of *UCP1*, a gene involved in thermogenesis and heat production [32, 33]. In particular, reduced expression of *PPARGC1A* in brown adipose tissue is associated with decreased thermogenic activity, which may contribute to increased cold sensitivity [34]. In addition, the *BOK* gene is essential for transferring Ca^2+^ from the endoplasmic reticulum to mitochondria [35], maintaining the activation of mitochondrial dehydrogenases crucial for energy production [36]. This suggests that the regulation of *BOK* could be linked to CP by affecting energy production.

The regulation of endothelial cell proliferation was also enriched in the CP. Endothelial cells, like blood vessels, play a critical role in regulating blood flow and angiogenesis [37]. Dysregulation of endothelial cell proliferation may narrow peripheral blood vessels, impair circulation, and increase susceptibility to cold [38]. In addition, the regulation of cardiac muscle relaxation is relevant to CP. Impaired relaxation can lead to reduced ventricular filling, lowering cardiac output and blood flow to peripheral tissues, which is a common feature of CP [39, 40].

In control traits for HP or CP, although the numbers and ratios of significant SNPs are similar to cold/heat and thyroid traits (Supporting Table 2), GSEA and pathway similarity analysis showed no relation to C-HP characteristics. These results confirmed the associations of cold/heat and thyroid traits with C-HPs. Furthermore, given the biological relevance of thyroid hormone function to thermoregulation and metabolism, these traits can serve as reliable surrogate markers for C-HP [41, 42].

Our PCA results, showing distinct population clustering, highlight potential variations in C-HP across populations [43]. Notably, AFR and EAS populations formed well-separated clusters, suggesting significant differences in their C-HP characteristics. The independent clustering of the EAS population further underscores unique cold-adaptive traits that may influence their C-HP. These traits, possibly linked to archaic human ancestry and prolonged exposure to cold climates [44], may contribute to population-specific variations in C-HP among EAS, AFR, and other populations.

Our study has some limitations. Due to the limited availability of research specific to C-HP in traditional medicine, we focused on traits related to temperature sensitivity and thyroid hormones as primary candidates and validated their relevance through a biological control analysis. However, our control analysis was limited to just four traits, which restricts our ability to fully assess specificity. Although thyroid function showed the highest combined SNP ratio (0.142 versus 0.132 for myopia), the difference is modest. In addition, the nonzero hit rates observed in control traits suggest that some degree of background signal remains despite our randomization procedure. Nevertheless, GSEA confirmed that the biological interpretations of the candidate SNPs were consistent with C-HP phenotypes, and most SNPs from thyroid hormone were not pleiotropic, suggesting their high potential as reliable markers for C-HP diagnosis (Supporting Table 3). Furthermore, pathway similarity analysis revealed distinct patterns between control traits and thyroid-related pathways, supporting the validity of cold/heat and thyroid hormone traits as appropriate proxies for C-HP characteristics. Second, the validation using in-house data had limited statistical power owing to the small sample size (*N* = 90), and multiple testing corrections were not applied. To address this, we performed residual permutation, which adjusts for covariates and yields a more reliable null distribution in small-sample settings. In addition, the strong interconnectivity was only observed with cold/heat and thyroid hormone traits in the pathway similarity network analysis after GSEA. Third, ethnic differences should be taken into account when interpreting our findings. Most previous GWAS have been conducted in EUR populations, which may have led to different alternative alleles being reported for the same SNPs compared to our data. In addition, effect sizes may vary across populations [45]. Therefore, larger and more diverse cohorts are essential to increase the statistical power of future studies and validate our findings on a broader scale. Functional studies are also needed to clarify how these genetic markers influence the biological pathways related to C-HP, such as mitochondrial regulation and vascular development. Finally, integrating multiomics approaches, including proteomics and metabolomics, into future research could provide a more comprehensive understanding of how gene expression and metabolite levels contribute to C-HP.

## 5. Conclusion

This study identified 39 candidate SNPs associated with C-HP, contributing to the limited pool of genetic biomarkers for C-HP in traditional medicine. Identification of these SNPs offers a valuable foundation for future biological research and clinical applications of C-HP. We hope that our findings will contribute to the development of more precise and individualized treatment strategies for patients based on their genetic makeup, helping bridge the gap between traditional medicine and modern precision medicine.

## Figures and Tables

**Table 1:** Candidate SNPs for heat-pattern traits.

	SNP	CHR	POS	Reported trait	Genes	Previous GWAS data			Replication in in-house data					
						BETA	A1	*P* value	A2	A1	MAF	BETA	SE	Permutation *p* value
Heat pattern–related thyroid hormone SNPs	rs12409301	1	19,448,221	Thyroid-stimulating hormone	*CAPZB*	−0.053	A	3 × 10^−78^	G	A	0.283	2.312	0.976	2.05 × 10^−02^
	rs12855	1	50,974,421	Thyroid-stimulating hormone	*CDKN2C*	0.033	T	3 × 10^−13^	C	T	0.144	−2.256	1.091	4.20 × 10^−02^
	rs951366	1	205,716,224	Thyroxine	*PM20D1*	0.037	T	6 × 10^−08^	T	C	0.272	−2.74	1.004	7.20 × 10^−03^
	rs59381142	3	194,198,392	Thyroid-stimulating hormone	*HES1*	−0.049	A	3 × 10^−53^	G	A	0.311	1.9	0.874	3.48 × 10^−02^
	rs11755845	6	43,937,043	Thyroid-stimulating hormone	*VEGFA*	−0.065	T	2 × 10^−10^	C	T	0.117	−2.5	1.223	4.45 × 10^−02^
	rs10814915	9	4,290,544	Thyroid-stimulating hormone	*GLIS3*	0.053	T	1 × 10^−95^	C	T	0.439	−1.772	0.767	2.29 × 10^−02^
	rs7045138	9	97,829,181	Thyroxine	*TRMO*	0.098	T	2 × 10^−11^	T	C	0.122	−3.221	1.24	1.19 × 10^−02^
	rs11255790	10	8,640,217	Thyroid-stimulating hormone	*NA*	−0.027	T	4 × 10^−21^	C	T	0.128	3.594	1.159	3.10 × 10^−03^
	rs10905868	10	10,932,293	Thyroid-stimulating hormone	*CELF2*	0.02	G	4 × 10^−06^	A	G	0.094	3.743	1.334	6.70 × 10^−03^
	rs4933466	10	88,089,762	Thyroid-stimulating hormone	*PTEN*	0.028	A	2 × 10^−26^	G	A	0.222	−2.149	1.018	4.30 × 10^−02^
	rs2475217	10	103,914,337	Triiodothyronine	*SLK*	0.019	C	3 × 10^−06^	C	T	0.011	8.125	3.785	3.37 × 10^−02^
	rs643506	11	111,765,903	Thyroid-stimulating hormone	*PPP2R1B*	−0.021	T	6 × 10^−14^	G	T	0.178	2.443	1.11	2.64 × 10^−02^
	rs71430783	14	93,092,108	Thyroid-stimulating hormone	*ITPK1*	0.069	T	2 × 10^−110^	G	T	0.089	2.791	1.391	4.70 × 10^−02^
	rs2601803	16	4,095,640	Thyroid-stimulating hormone	*ADCY9*	−0.028	C	4 × 10^−13^	C	G	0.433	2.489	0.873	7.10 × 10^−03^
	rs3848445	17	14,390,704	Triiodothyronine	*HS3ST3B1*	—	—	8 × 10^−09^	T	C	0.361	−2.317	0.789	4.20 × 10^−03^
	rs75261749	17	51,582,013	Thyroid-stimulating hormone	*UTP18*	−1.421	A	9 × 10^−24^	G	A	0.033	7.106	2.193	2.40 × 10^−03^
	rs9915657	17	72,131,395	Thyroid-stimulating hormone	*SOX9*	−0.064	T	8 × 10^−13^	C	T	0.239	2.425	0.947	1.30 × 10^−02^
	rs4552110	18	59,516,716	Thyroid-stimulating hormone	*CCBE1*	−0.0303	A	1 × 10^−19^	A	T	0.0555	−4.167	1.803	2.12 × 10^−02^

Heat pattern–related cold/heat SNPs	rs62143197	19	53,817,462	Cold-inducible RNA-binding protein	*NLRP12*	0.66	A	4 × 10^−120^	G	A	0.133	−2.943	1.431	4.31 × 10^−02^
	rs4666462	2	19,117,243	Response to cold	*OSR1*	—	—	2 × 10^−06^	A	G	0.133	2.606	1.271	4.45 × 10^−02^

**Table 2:** Candidate SNPs for cold-pattern traits.

	SNPS	CHR	POS	Reported trait	Genes	Previous GWAS data			Replication in in-house data					
						BETA	A1	*P* value	A2	A1	MAF	BETA	SE	Permutation *p* value
Cold pattern–related thyroid hormone SNPs	rs12855	1	50,974,421	Thyroid-stimulating hormone	*CDKN2C*	0.033	T	3 × 10^−13^	C	T	0.144	2.236	1.07	3.90 × 10^−02^
	rs622474	1	66,277,601	Thyroid-stimulating hormone	*PDE4B*	0.02	A	8 × 10^−15^	A	G	0.483	1.692	0.759	2.70 × 10^−02^
	rs11204752	1	150,985,657	Thyroid-stimulating hormone	*GOLPH3L*	0.017	T	2 × 10^−10^	C	T	0.367	1.782	0.816	3.08 × 10^−02^
	rs6722076	2	233,738,671	Thyroxine	*UGT1A6*	−0.104	A	6 × 10^−10^	G	A	0.15	2.621	1.044	1.30 × 10^−02^
	rs6717283	2	241,576,690	Thyroid-stimulating hormone	*BOK*	0.046	G	1 × 10^−11^	A	G	0.078	3.888	1.528	1.41 × 10^−02^
	rs16874919	4	24,063,586	Thyroid-stimulating hormone	*PPARGC1A*	0.025	A	2 × 10^−17^	G	A	0.283	−2.095	1.006	3.85 × 10^−02^
	rs6462411	7	3,875,932	Thyroid-stimulating hormone	*SDK1*	−0.36	C	1 × 10^−06^	T	C	0.156	−2.456	1.135	3.51 × 10^−02^
	rs59282311	8	65,974,221	Thyroid-stimulating hormone	*PDE7A*	−0.019	A	1 × 10^−12^	A	G	0.111	2.538	1.263	4.81 × 10^−02^
	rs1441198	8	69,506,886	Thyroid-stimulating hormone	*SULF1*	0.018	A	1 × 10^−11^	A	G	0.1	−3.092	1.361	2.63 × 10^−02^
	rs9298749	9	16,214,342	Thyroid-stimulating hormone	*BNC2*	−0.038	A	6 × 10^−11^	C	A	0.35	1.874	0.831	2.54 × 10^−02^
	rs925489	9	97,784,318	Thyroid-stimulating hormone	*TRMO*	−0.058	C	1 × 10^−13^	T	C	0.044	3.787	1.769	3.41 × 10^−02^
	rs7855088	9	97,979,971	Thyroid-stimulating hormone	*ANP32B*	−0.05	C	6 × 10^−06^	C	T	0.339	2.249	1.018	2.77 × 10^−02^
	rs657152	9	133,263,862	Thyroid-stimulating hormone	*ABO*	0.058	A	4 × 10^−10^	C	A	0.422	−1.747	0.795	2.93 × 10^−02^
	rs7020640	9	136,653,172	Triiodothyronine	*EGFL7*	0.026	T	9 × 10^−07^	C	T	0.083	4.245	1.338	2.80 × 10^−03^
	rs6499766	16	55,570,216	Thyroxine	*LPCAT2*	0.056	A	1 × 10^−06^	T	A	0.328	1.847	0.808	2.27 × 10^−02^
	rs7253430	19	1,803,583	Thyroid-stimulating hormone	*ATP8B3*	0.02	A	7 × 10^−06^	G	T	0.272	−2.112	0.965	3.93 × 10^−02^

Cold pattern–related cold/heat SNPs	rs1354034	3	56,815,721	Cold-inducible RNA-binding protein	*ARHGEF3*	−0.183	T	4 × 10^−46^	C	T	0.444	2.008	0.757	1.80 × 10^−03^
	rs17122904	1	62,006,470	Cold-induced vasodilation	*PATJ*	—	—	8 × 10^−06^	A	G	0.406	−1.679	0.831	4.87 × 10^−02^
	rs77101060	10	106,633,287	Cold sensitivity	*SORCS1*	−0.96	T	6.45 × 10^−06^	C	T	0.261	1.948	0.913	3.54 × 10^−02^

## Data Availability

Publicly available data from the GWAS Catalog (EMBL-EBI) were used for this study and can be accessed at https://www.ebi.ac.uk/gwas/. The 1000 Genomes Project reference dataset is also available at https://ftp.1000genomes.ebi.ac.uk/vol1/ftp/release/20130502/. The in-house data that were used to validate the findings are available from the corresponding author upon reasonable request.

## Supporting Information

Supporting Information 1 Supporting Table 1: Full names of genes associated with candidate SNPs. Supporting Table 2: SNP and gene summary for C-HPs. Supporting Table 3: GWAS catalog traits associated with C-HPs candidate SNPs.

Supporting Information 2 Supporting Figure 1: *p* values of reported SNPs for thyroid hormone and cold/heat-related traits. Histograms show the distribution of 128 SNPs associated with cold/heat-related traits (A) and 479 SNPs associated with thyroid hormone traits (B) selected from the GWAS catalog and literature. The *X*-axis represents -log10 (*p* value), and the *Y*-axis represents the number of SNPs.

Supporting Information 3 Supporting Figure 2: Pathway similarity network analysis for HP. The top 5 enriched pathways from each trait were visualized as nodes, with node size proportional to –log10 (*p* value) from GSEA for HP. Edges represent pathway similarity quantified by the Jaccard index, and edge thickness corresponds to the degree of gene overlap. Results are shown for (A) cold/heat and thyroid, (B) body height, (C) hair color, and (D) myopia.

Supporting Information 4 Supporting Figure 3: Pathway similarity network analysis for CP. The top 5 enriched pathways from each trait were visualized as nodes, with node size proportional to –log10 (*p* value) from GSEA for CP. Edges represent pathway similarity quantified by the Jaccard index, and edge thickness corresponds to the degree of gene overlap. Results are shown for (A) cold/heat and thyroid, (B) body height, (C) hair color, and (D) myopia.

Supporting Information 5 Supporting Figure 4: GSEA results for control traits in HP. GSEA was performed on genes mapped to SNPs with permutation *p* value < 0.05 from association tests with the HP phenotype for three control traits: (A) body height, (B) hair color, and (C) myopia. Bar plots show the top 5 pathways with the lowest *p* values. No pathways showed biologically significant enrichment related to C-HPs.

Supporting Information 6 Supporting Figure 5: GSEA results for control traits in CP. GSEA was performed on genes mapped to SNPs with permutation *p* value < 0.05 from association tests with the CP phenotype for three control traits: (A) body height, (B) hair color, and (C) myopia. Bar plots show the top 5 pathways with the lowest *p* values. No pathways showed biologically significant enrichment related to C-HPs.
