# Supplementary material for: Exploring Genetic Markers for Cold–Heat Patterns: Integrating Traditional Medicine With Modern Genomic Research
Source: Genet Res (Camb). 2025 Nov 21;2025:4503515. doi: 10.1155/genr/4503515 (PMC12662677; doi:10.1155/genr/4503515)
Supplement: Supporting Information 1 — Supporting Table 1: Full names of genes associated with candidate SNPs. Supporting Table 2: SNP and gene summary for C-HPs. Supporting Table 3: GWAS catalog traits associated with C-HPs candidate SNPs. [file 4503515.f1.docx]

| **Genes** | **Full names of the candidate genes** |
| --- | --- |
| *CAPZB* | Capping Actin Protein Of Muscle Z-Line Subunit Beta |
| *CDKN2C* | Cyclin Dependent Kinase Inhibitor 2C |
| *PM20D1* | Peptidase M20 Domain Containing 1 |
| *HES1* | Hes Family BHLH Transcription Factor 1 |
| *VEGFA* | Vascular Endothelial Growth Factor A |
| *GLIS3* | GLIS Family Zinc Finger 3 |
| *TRMO* | TRNA Methyltransferase O |
| *CELF2* | CUGBP Elav-Like Family Member 2 |
| *PTEN* | Phosphatase And Tensin Homolog |
| *SLK* | STE20 Like Kinase |
| *PPP2R1B* | Protein Phosphatase 2 Scaffold Subunit Abeta |
| *ITPK1* | Inositol-Tetrakisphosphate 1-Kinase |
| *ADCY9* | Adenylate Cyclase 9 |
| *HS3ST3B1* | Heparan Sulfate-Glucosamine 3-Sulfotransferase 3B1 |
| *UTP18* | UTP18 Small Subunit Processome Component |
| *SOX9* | SRY-Box Transcription Factor 9 |
| *CCBE1* | Collagen And Calcium Binding EGF Domains 1 |
| *NLRP12* | NLR Family Pyrin Domain Containing 12 |
| *OSR1* | Odd-Skipped Related Transcription Factor 1 |
| *PDE4B* | Phosphodiesterase 4B |
| *GOLPH3L* | Golgi Phosphoprotein 3 Like |
| *BOK* | BCL2 Family Apoptosis Regulator BOK |
| *UGT1A6* | UDP Glucuronosyltransferase Family 1 Member A6 |
| *PPARGC1A* | PPARG Coactivator 1 Alpha |
| *SDK1* | Sidekick Cell Adhesion Molecule 1 |
| *SULF1* | Sulfatase 1 |
| *PDE7A* | Phosphodiesterase 7A |
| *EGFL7* | EGF Like Domain Multiple 7 |
| *BNC2* | Basonuclin Zinc Finger Protein 2 |
| *ANP32B* | Acidic Nuclear Phosphoprotein 32 Family Member B |
| *ABO* | Alpha 1-3-N-Acetylgalactosaminyltransferase And Alpha 1-3-Galactosyltransferase |
| *LPCAT2* | Lysophosphatidylcholine Acyltransferase 2 |
| *ATP8B3* | ATPase Phospholipid Transporting 8B3 |
| *ARHGEF3* | Rho Guanine Nucleotide Exchange Factor 3 |
| *PATJ* | PATJ Crumbs Cell Polarity Complex Component |
| *SORCS1* | Sortilin Related VPS10 Domain Containing Receptor 1 |

**Supporting Table 1. Full names of genes associated with candidate SNPs.**

**Supporting Table 2. SNP and Gene Summary for Heat and Cold Patterns (C-HP)**

SNPs indicate variants after excluding those in linkage disequilibrium (LD). Significant SNPs refer to those with permutation P-values < 0.05 obtained from residual permutation testing. The ratio indicates the proportion of significant SNPs among total SNPs, and genes represent those mapped to significant SNPs.

|  |  | **Cold/Heat and Thyroid** | **Body height** | **Hair color** | **Myopia** |
| --- | --- | --- | --- | --- | --- |
| **Heat Pattern** | **SNPs** | 271 | 476 | 197 | 319 |
|  | **Significant SNPs** | 20 | 24 | 15 | 18 |
|  | **Ratio** | 0.074 | 0.05 | 0.076 | 0.056 |
|  | **Genes** | 19 | 12 | 15 | 14 |
| **Cold Pattern** | **SNPs** | 278 | 476 | 196 | 317 |
|  | **Significant SNPs** | 19 | 27 | 11 | 24 |
|  | **Ratio** | 0.068 | 0.057 | 0.056 | 0.076 |
|  | **Genes** | 19 | 19 | 11 | 21 |
| **Total** | **SNPs** | 549 | 952 | 393 | 636 |
|  | **Significant SNPs** | 39 | 51 | 26 | 42 |
|  | **Ratio** | 0.142 | 0.107 | 0.132 | 0.132 |
|  | **Genes** | 38 | 31 | 26 | 35 |

**Supporting Table 3. GWAS catalog traits associated with C-HP candidate SNPs**

| **Cold-pattern candidate SNP** | **Genes** | **GWAS catalog traits** |
| --- | --- | --- |
| rs622474 | *PDE4B* | thyroid stimulating hormone amount |
| rs11204752 | *GOLPH3L* | neuroblastoma, cutaneous melanoma, tonsillitis, thyroid stimulating hormone amount |
| rs12855 | *CDKN2C* | body height, type 2 diabetes mellitus, thyroid stimulating hormone amount, body height |
| rs6717283 | *BOK* | thyroid stimulating hormone amount |
| rs6722076 | *UGT1A6* | bilirubin measurement, insomnia, xanthurenate measurement, thyroxine amount |
| rs16874919 | *PPARGC1A* | thyroid stimulating hormone amount |
| rs6462411 | *SDK1* | thyroid stimulating hormone amount |
| rs1441198 | *SULF1* | thyroid stimulating hormone amount |
| rs59282311 | *PDE7A* | erythrocyte count, thyroid stimulating hormone amount |
| rs7020640 | *EGFL7* | thyroxine amount, triiodothyronine measurement |
| rs9298749 | *BNC2* | thyroid stimulating hormone amount, hypothyroidism |
| rs7855088 | *ANP32B* | thyroid stimulating hormone amount |
| rs657152 | *ABO* | estradiol measurement, hormone measurement, thyroid stimulating hormone amount, level of integrin alpha-11 in blood serum, etc |
| rs925489 | *TRMO* | hypothyroidism, autoimmune thyroid disease, thyroid stimulating hormone amount, thyroid cancer, thyroid carcinoma,atrial fibrillation |
| rs544873 | *ABO* | thyroid stimulating hormone amount |
| rs6499766 | *LPCAT2* | thyroxine amount, hormone measurement |
| rs7253430 | *ATP8B3* | thyroid stimulating hormone amount |
| rs1354034 | *ARHGEF3* | Cold-inducible RNA-binding, protein, protein measurement, blood protein amount, etc |
| rs17122904 | *PATJ* | cold-induced vasodilation |
| rs77101060 | *SORCS1* | Cold sensitivity |
