# Supplementary figures and images for: Exploring Genetic Markers for Cold–Heat Patterns: Integrating Traditional Medicine With Modern Genomic Research
Source: Genet Res (Camb). 2025 Nov 21;2025:4503515. doi: 10.1155/genr/4503515 (PMC12662677; doi:10.1155/genr/4503515)

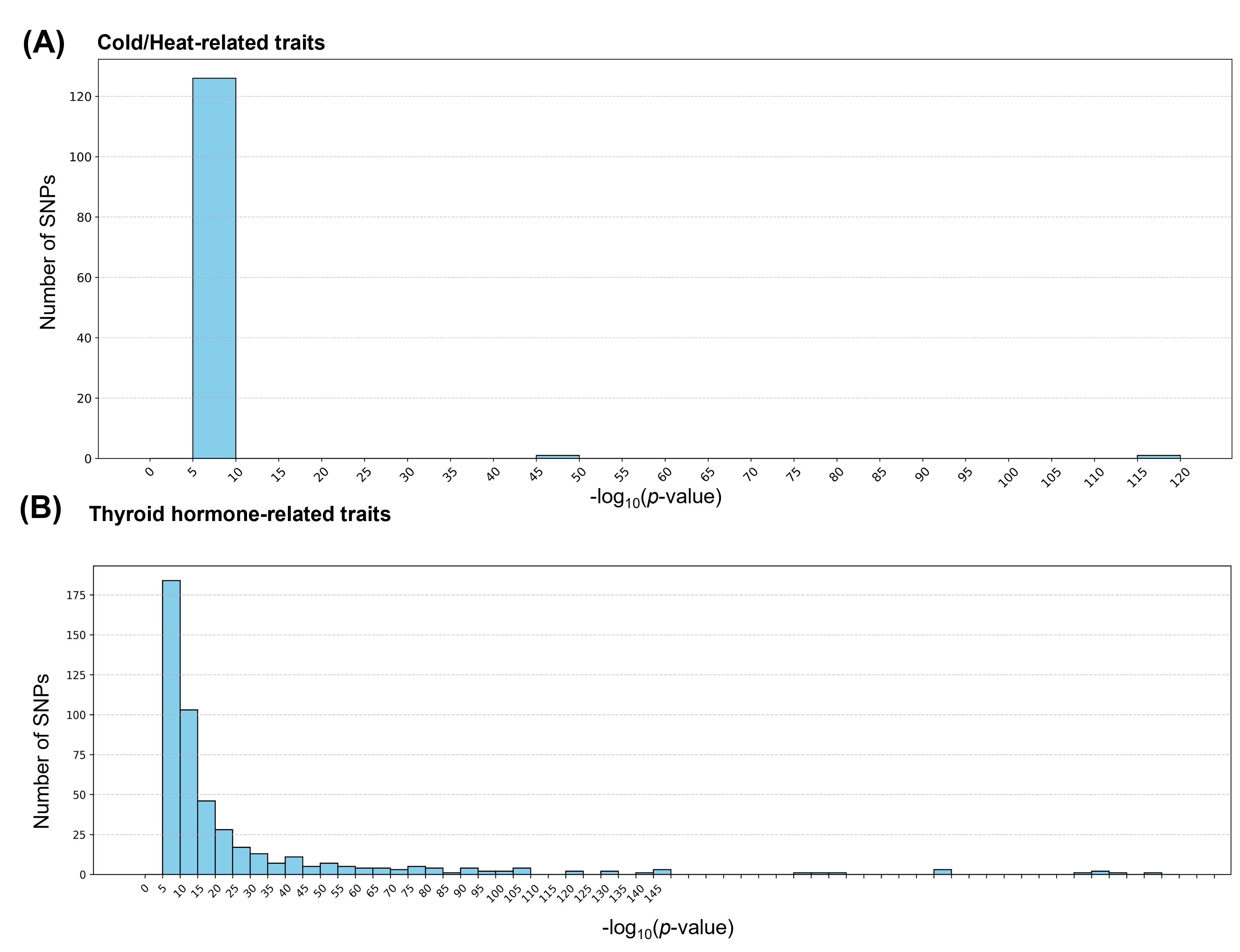

Supplement: Supporting Information 2 — Supporting Figure 1: p values of reported SNPs for thyroid hormone and cold/heat-related traits. Histograms show the distribution of 128 SNPs associated with cold/heat-related traits (A) and 479 SNPs associated with thyroid hormone traits (B) selected from the GWAS catalog and literature. The X-axis represents -log10 (p value), and the Y-axis represents the number of SNPs. [file 4503515.f2.tif]

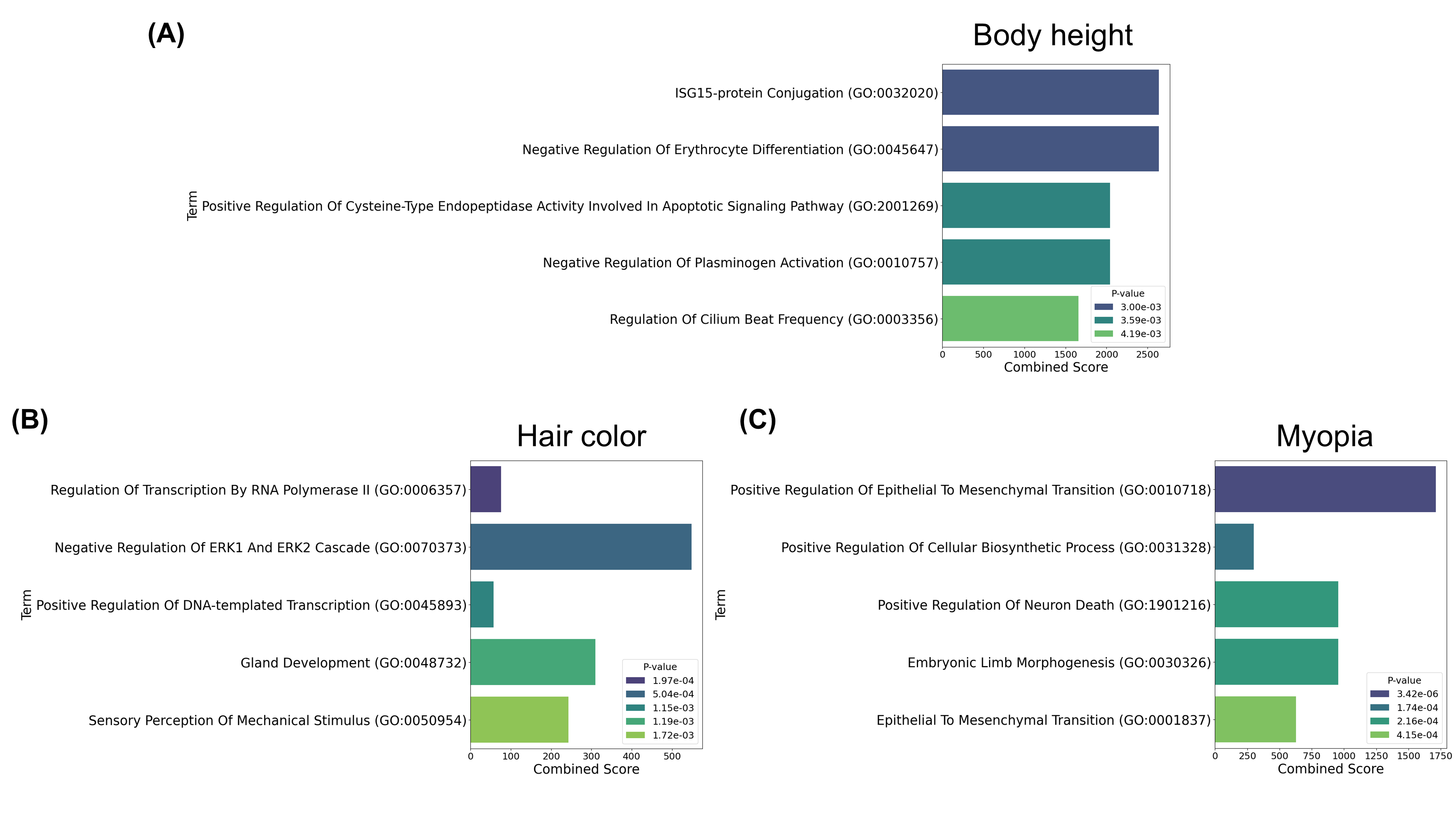

Supplement: Supporting Information 3 — Supporting Figure 2: Pathway similarity network analysis for HP. The top 5 enriched pathways from each trait were visualized as nodes, with node size proportional to –log10 (p value) from GSEA for HP. Edges represent pathway similarity quantified by the Jaccard index, and edge thickness corresponds to the degree of gene overlap. Results are shown for (A) cold/heat and thyroid, (B) body height, (C) hair color, and (D) myopia. [file 4503515.f3.tif]

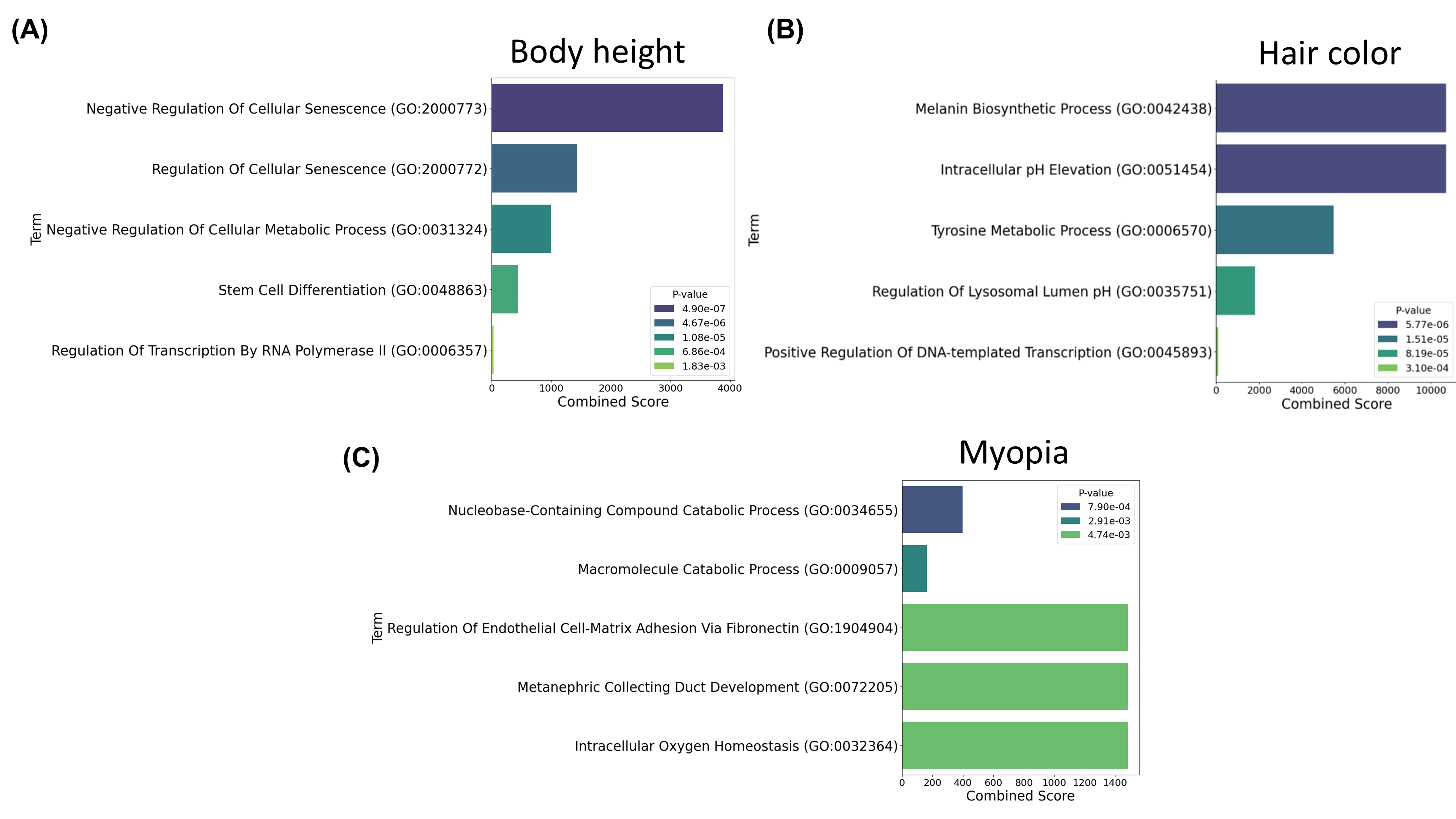

Supplement: Supporting Information 4 — Supporting Figure 3: Pathway similarity network analysis for CP. The top 5 enriched pathways from each trait were visualized as nodes, with node size proportional to –log10 (p value) from GSEA for CP. Edges represent pathway similarity quantified by the Jaccard index, and edge thickness corresponds to the degree of gene overlap. Results are shown for (A) cold/heat and thyroid, (B) body height, (C) hair color, and (D) myopia. [file 4503515.f4.tif]

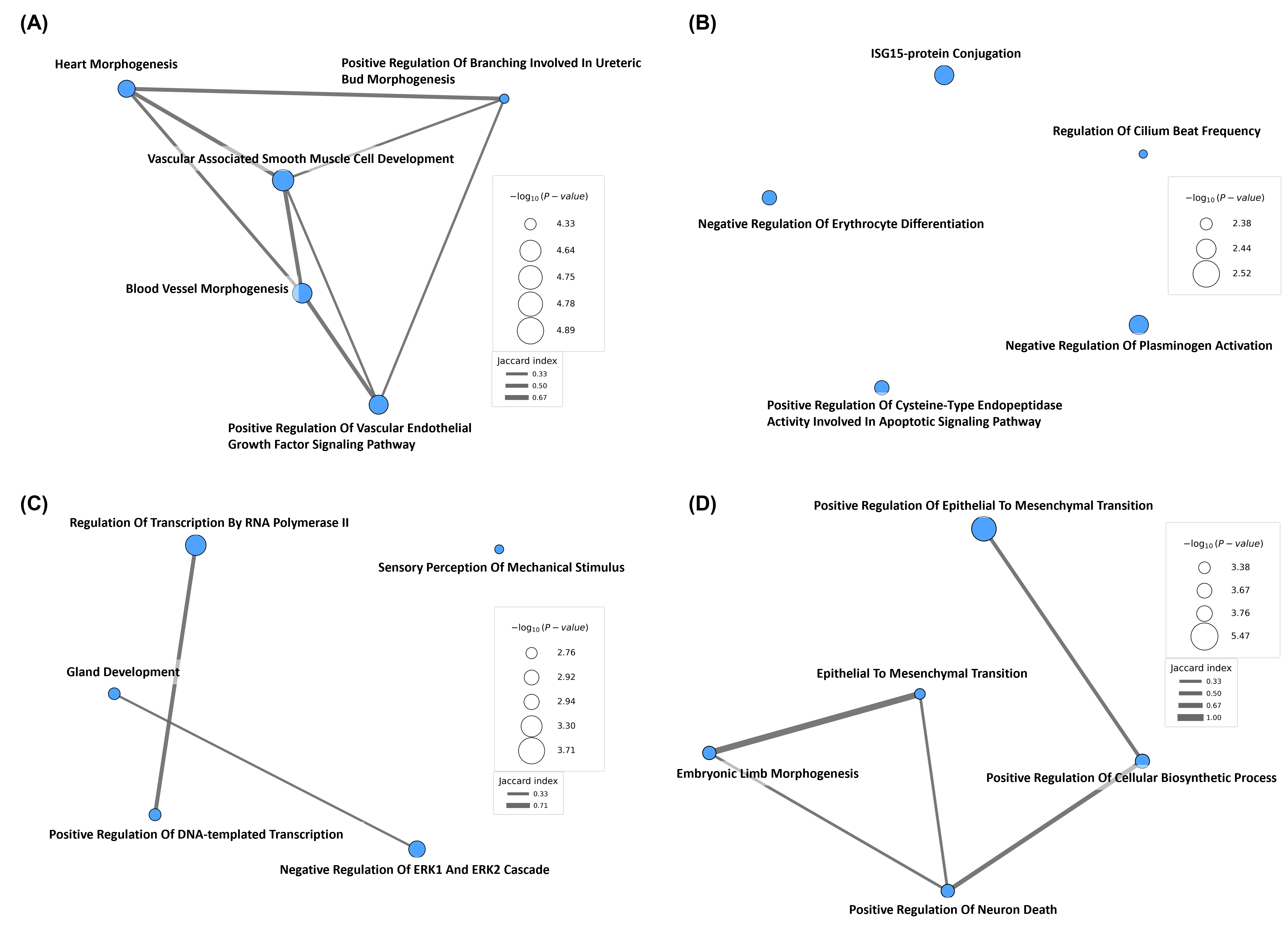

Supplement: Supporting Information 5 — Supporting Figure 4: GSEA results for control traits in HP. GSEA was performed on genes mapped to SNPs with permutation p value < 0.05 from association tests with the HP phenotype for three control traits: (A) body height, (B) hair color, and (C) myopia. Bar plots show the top 5 pathways with the lowest p values. No pathways showed biologically significant enrichment related to C-HPs. [file 4503515.f5.tif]

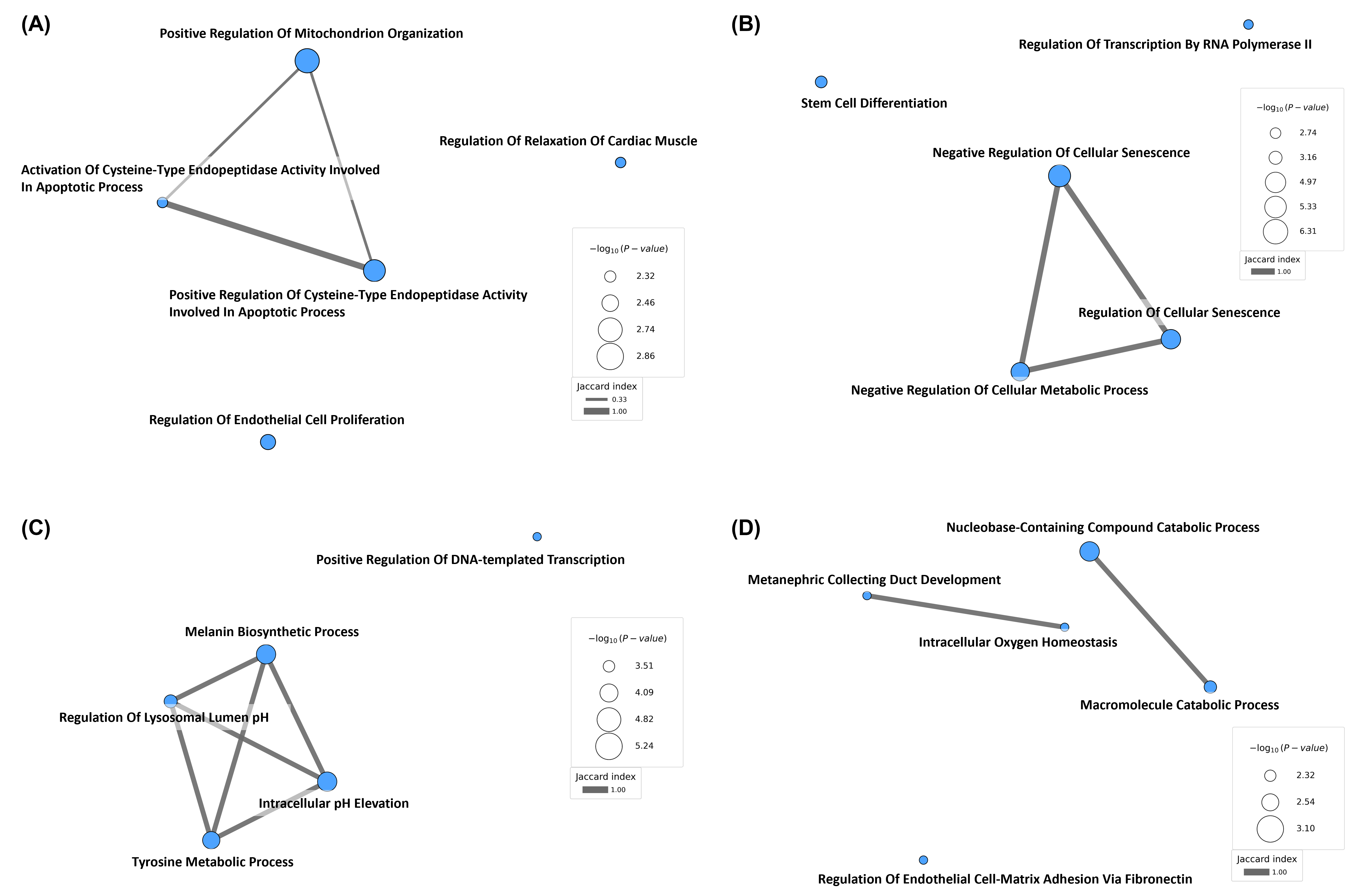

Supplement: Supporting Information 6 — Supporting Figure 5: GSEA results for control traits in CP. GSEA was performed on genes mapped to SNPs with permutation p value < 0.05 from association tests with the CP phenotype for three control traits: (A) body height, (B) hair color, and (C) myopia. Bar plots show the top 5 pathways with the lowest p values. No pathways showed biologically significant enrichment related to C-HPs. [file 4503515.f6.tif]
